# Advancement in Cancer Vasculogenesis Modeling through 3D Bioprinting Technology

**DOI:** 10.3390/biomimetics9050306

**Published:** 2024-05-20

**Authors:** Arvind Kumar Shukla, Sik Yoon, Sae-Ock Oh, Dongjun Lee, Minjun Ahn, Byoung Soo Kim

**Affiliations:** 1 School of Biomedical Convergence Engineering, Pusan National University, Yangsan 50612, Republic of Korea; 2 Department of Anatomy and Convergence Medical Sciences, Pusan National University College of Medicine, Yangsan 50612, Republic of Korea; 3 Immune Reconstitution Research Center of Medical Research Institute, Pusan National University College of Medicine, Yangsan 50612, Republic of Korea; 4 Research Center for Molecular Control of Cancer Cell Diversity, Pusan National University, Yangsan 50612, Republic of Korea; 5 Department of Anatomy, School of Medicine, Pusan National University, Yangsan 50612, Republic of Korea; 6 Department of Convergence Medicine, Pusan National University College of Medicine, Yangsan 50612, Republic of Korea; 7 Medical Research Institute, Pusan National University, Yangsan 50612, Republic of Korea

**Keywords:** 3D bioprinting technology, cancer, vasculogenesis, cancer modeling, cancer microenvironment

## Abstract

Cancer vasculogenesis is a pivotal focus of cancer research and treatment given its critical role in tumor development, metastasis, and the formation of vasculogenic microenvironments. Traditional approaches to investigating cancer vasculogenesis face significant challenges in accurately modeling intricate microenvironments. Recent advancements in three-dimensional (3D) bioprinting technology present promising solutions to these challenges. This review provides an overview of cancer vasculogenesis and underscores the importance of precise modeling. It juxtaposes traditional techniques with 3D bioprinting technologies, elucidating the advantages of the latter in developing cancer vasculogenesis models. Furthermore, it explores applications in pathological investigations, preclinical medication screening for personalized treatment and cancer diagnostics, and envisages future prospects for 3D bioprinted cancer vasculogenesis models. Despite notable advancements, current 3D bioprinting techniques for cancer vasculogenesis modeling have several limitations. Nonetheless, by overcoming these challenges and with technological advances, 3D bioprinting exhibits immense potential for revolutionizing the understanding of cancer vasculogenesis and augmenting treatment modalities.

## 1. Introduction

Vascularization refers to the process of forming blood vessels within tissues or organs and involves the growth and development of new blood vessels, including arteries, veins, and capillaries [1,2]. Each type of blood vessel has specific characteristics and functions. Arteries carry oxygen-rich blood away from the heart to various parts of the body, featuring thick, muscular walls composed of smooth muscle fibers and elastic tissue, enabling them to withstand high blood pressure and regulate blood flow. Veins, on the other hand, transport oxygen-depleted blood back to the heart, with thinner walls compared to arteries and containing valves to prevent the backflow of blood [3,4,5]. Capillaries, the smallest blood vessels in the body, form an extensive network within tissues, facilitating the exchange of gases, nutrients, and waste products between the blood and surrounding cells [6,7]. In addition to their functionality, vascular networks comprise a complex geometry and are closely related to intricate interactions between different cell–cell, cell–extracellular matrix (ECM), and cell-signaling molecules [8,9]. Replicating the complexity and physiological characteristics of blood vessels in vitro, including processes such as angiogenesis, vascular remodeling, and adaptability to physiological signals, is a significant challenge [10,11]. Establishing this level of realism involves a complex process at the cutting edge of tissue engineering and regenerative medicine research, requiring innovative approaches in biomaterials engineering, advanced biofabrication techniques, and an in-depth understanding of vascular biology [12,13,14].

Cancer vasculogenesis is essential for tumor development, metastasis, and the creation of a vasculogenic environment that supports cancer growth and is a central area of study and therapy for cancers [15]. However, the complexities of this phenomenon pose significant challenges to precise modeling of the complex microenvironment, which is essential for understanding cancer development when investigated using traditional investigative techniques [16,17]. More recently, advancements in three-dimensional (3D) bioprinting present promising ways to overcome these limitations [18,19,20]. To develop cancer vasculogenesis models, this study aims to provide an in-depth understanding of the subject, emphasize the significance of appropriate modeling, and compare conventional methods with the cutting-edge potential of 3D bioprinting technology [16,21,22,23]. Recent advancements of 3D bioprinting offers a promising solution to conventional hurdles such as the formation of stable and perfusable blood vessels in vitro, as it enables precise control over the spatial arrangement of cells, biomaterials, and signaling molecules [24,25]. Moreover, bioprinting techniques allow for the incorporation of support structures and bioactive factors to promote vascularization and tissue integration. This capability is crucial for developing functional tissue constructs with physiologically relevant vascularization, paving the way for applications such as organ-on-a-chip platforms, drug screening assays, and, ultimately, tissue replacement therapies. As we delve deeper into the discussion on vascularization and tissue engineering, the role of 3D bioprinting emerges as a transformative tool in overcoming the complexities inherent in replicating the intricate structures of the human vasculature.

Historically, approaches that frequently failed to adequately observe the complex interactions that occur within tumor microenvironments have been used by researchers to address the complexity of cancer vasculogenesis [26,27,28]. The shortcomings of these methods highlight the pressing demand for more advanced techniques that accurately replicate the complex interactions between cancer cells, vascular networks, and surrounding tissues [29,30,31]. Under these conditions, 3D bioprinting is a revolutionary technique that provides previously unattainable levels of precision and control to develop complex tissue structures that closely mimic natural physiological conditions [32,33,34,35]. To develop targeted therapeutic methods for preventing tumor development and metastasis, a thorough understanding of cancer vasculogenesis is crucial. The complexities of tumor microenvironments have proven challenging to understand using conventional techniques [16,17]. However, the field has completely changed with the development of 3D bioprinting technology, which provides an entirely novel technique for in vitro replication of the intricate architecture of tumors [36,37,38,39]. This development may lead to the discovery of novel treatment approaches to stop tumor angiogenesis and the spread of metastatic disease by providing insights into the fundamental principles of cancer vasculogenesis [36,37,40].

Generally, it is difficult to accurately replicate complex vascular networks signifying malignant tumors using conventional methods. Because of these limitations, it is challenging for researchers to precisely understand the complexities of cancer vasculogenesis [30,41,42]. However, by leveraging 3D bioprinting, researchers can now create complex in vitro models that accurately replicate the 3D architecture of tumor microenvironments [43,44,45]. By carefully coordinating cellular, biomaterial, and growth factor deposition, complex vascular networks can be created in these models that closely resemble the complexity observed in vivo [14,17,46,47,48]. Researchers can now fully explore the mechanisms underlying cancer vasculogenesis, thereby providing significant insights into the mechanisms underlying tumor angiogenesis and metastasis [40,49,50,51]. This review explores several 3D bioprinting approaches aimed at creating customized patient-derived spheroids and organoids that mimic the complexity of cells in vitro. The emergence of 3D bioprinting technology has resulted in an important advancement in the development of vascular models that closely replicate the complex vessel networks observed within actual cancers.

**Figure 1:**
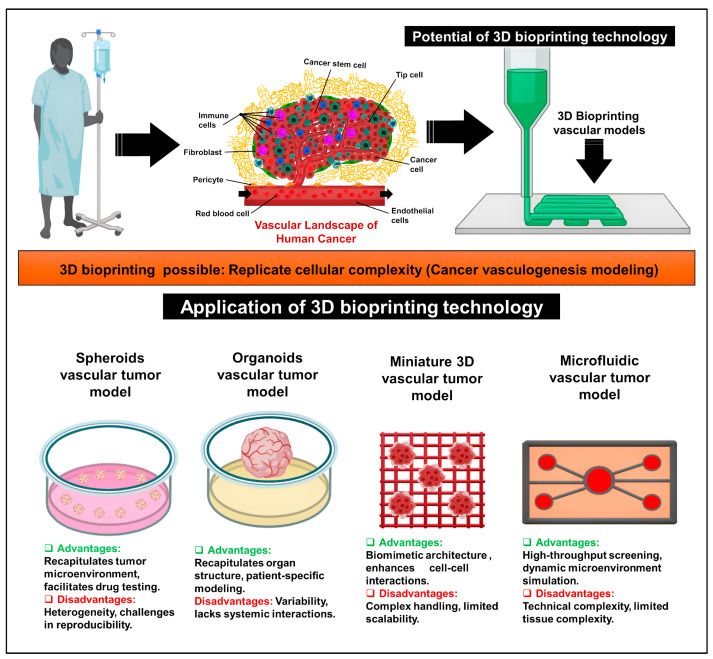
Application of 3D bioprinting technology for cancer vascular models. These technologies include a spheroid vascular tumor model, organoid vascular tumor model, miniature 3D vascular tumor model, and microfluidic vascular tumor model.

This innovative 3D bioprinting technology makes it possible to replicate the cellular complexity in cancer vascular models using different techniques, each with specific advantages and disadvantages. These techniques include the spheroid vascular tumor model, organoid vascular tumor model, miniature 3D vascular tumor model, and microfluidic vascular tumor model (Figure 1). For example, spheroid vascular tumor models, a crucial tool in cancer research, effectively replicate the complex tumor environment. These models, by providing a more physiologically suitable environment, enable the evaluation of drug toxicity and efficacy. However, the challenges of heterogeneity and reproducibility, which are significant and complex, may limit their application in certain conditions. Despite these challenges, their importance in understanding tumor biology and developing treatment methods remains [52,53].

Organoid vascular tumor models, which accurately replicate the structural complexity of organs, provide an effective method closer to personalized therapy. This methodology makes patient-specific modeling and personalized therapeutic strategies possible. However, the lack of systemic interactions and heterogeneity in organoid models may limit their application in understanding complex tumor–host interactions. It is essential to maintain a balance between these advantages and disadvantages in order to utilize organoid models in cancer research effectively. Organoid vascular tumor models, which accurately replicate the structural complexity of organs, provide an effective method closer to personalized therapy. This methodology makes patient-specific modeling and personalized therapeutic strategies possible. However, the lack of systemic interactions and heterogeneity in organoid models may limit their application in understanding complex tumor–host interactions. Maintaining a balance between these advantages and disadvantages is essential to utilizing organoid models effectively in cancer research [54,55]. 

Miniature 3D vascular tumor models enhanced with vascular cells demonstrate advanced cellular interactions and biomimetic design. These models allow researchers to investigate complex cell-to-cell communication within a regulated microenvironment. However, their handling and scalability challenges could prevent them from being widely used in high-throughput applications. Miniature 3D models, despite these limitations, provide a significant understanding of tumor angiogenesis and therapeutic responses [56,57]. Microfluidic vascular tumor models, enabling high-throughput screening, have revolutionized the field of cancer research. These models, pivotal in precision medicine techniques, provide unique knowledge about tumor development and treatment responses. However, the challenges of reproducing tissue complexity and overcoming technological barriers persist, underscoring the urgent need for continuous innovation in microfluidic model development. To fully utilize the potential of 3D bioprinting in advancing cancer research and treatment paradigms, a thorough understanding of the complexities of each technique is essential [58,59]. 

The fabrication of 3D bioprinted cancer vasculogenesis models signifies an essential transformation in several fields associated with cancer research and therapeutic applications. The most prominent of these is preclinical drug screening, where conventional two-dimensional cell cultures have been extensively utilized [60,61,62]. However, a substantial percentage of clinical trial failures result from the frequent difficulty of these models in appropriately estimating the efficacy of drugs and their toxicity in vivo [63]. In contrast, 3D bioprinted tumor models provide a more physiologically appropriate platform for evaluating novel candidates for therapeutic purposes [63,64,65,66,67]. Researchers can measure medication responses more accurately by embedding tumor cells in a 3D matrix that replicates the natural tumor microenvironment. This increases the possibility of successfully translating the results into clinical settings [68,69,70]. More importantly, 3D bioprinted tumor models demonstrate significant potential for developing specific cancer diagnoses [71,72]. Conventional diagnostic methods frequently fail to consider the specific biological signals of individual cancers for generic biomarkers and histological analyses. Even by combining patient-derived cells into 3D bioprinted tumor models, researchers can customize diagnostic tests for the unique genetic markers of different cancers. With the significant potential to clarify individual therapeutic reactions and direct treatment decisions, this personalized approach may eventually lead to enhanced patient outcomes [73,74,75].

For example, vasculogenesis modeling has become a significant benefit in cancer studies, providing information about the dynamics of the environment and cancer angiogenesis. Three-dimensional bioprinted breast cancer models have addressed the vasculature’s function in treatment response and tumor growth. These models help accelerate the development of more effective therapies by precisely modeling the complex interactions between blood vessels and cancer cells. However, there are still challenges to replicating the diversity of breast cancers and the surrounding environments, limiting the predictive models’ application [76,77]. Vasculogenesis modeling in pancreatic cancer has provided beneficial methods for investigating drug resistance mechanisms and tumor–stromal interactions. Three-dimensional bioprinted models have helped researchers investigate the dynamic interactions between cancerous and endothelial cells by incorporating different cell types and extracellular matrix components. The complicated malignant microenvironment of pancreatic cancer is still challenging to fully show despite these developments due to challenges such as restricted vascularization and the absence of immune cell infiltration. Vasculogenesis modeling has shown the complex interactions between tumor cells and the surrounding vasculature in pancreatic cancer. The study of hypoxia-induced angiogenesis and the development of cutting-edge therapeutic strategies that target tumor angiogenesis have been made possible by 3D bioprinted models. Considering these developments, it is still challenging for researchers to find appropriate biomaterials that replicate the microenvironment of pancreatic tumors, which restricts the applicability of findings from studies into clinical application [78,79].

Vasculogenesis modeling in glioblastoma has been beneficial in understanding the highly angiogenic characteristics of these tumors. Using patient-derived cells in three-dimensional bioprinted models, researchers have effectively replicated the vascular mimicking observed in vivo. This approach offers the advantage of maintaining the genetic and phenotypic characteristics of the original tumor, enhancing the model’s relevance to clinical scenarios. However, challenges remain in precisely predicting clinical responses and maintaining the vascular network’s stability and functionality over extended culture times. The use of patient-derived cells also presents limitations, such as the difficulty in obtaining a sufficient number of cells and the potential for genetic drift during culture. In conclusion, while vasculogenesis modeling can enhance our understanding of cancer biology and treatment, it is crucial to overcome these challenges to broaden the application of the model to various cancer types and tissues [78,80].

Furthermore, the application of 3D bioprinted tumor models in pathological studies is expected to transform the current understanding of how cancer progresses and how resistance to treatment develops. Conventional histological examinations provide essential information about the physical characteristics of tumors but do not provide much information about the dynamic interactions between cells or the stimuli present in the microenvironment. In contrast, 3D bioprinted tumor models provide a more comprehensive understanding of tumor biology by enabling researchers to examine the complex interactions between tumor cells, stromal components, and the extracellular matrix. By analyzing these intricate relationships, scientists can find new therapeutic targets and create more potent treatment plans to halt the spread of cancer and overcome drug resistance [67,81]. 

In conclusion, 3D bioprinting technology is an innovative technique that can help understand the complex mechanisms involved in cancer vasculogenesis and advance the field of personalized cancer therapy. These models provide an outstanding understanding of the mechanisms underlying tumor angiogenesis and metastasis by precisely modeling the complex structure of the tumor microenvironment. They also show remarkable potential to transform personalized cancer diagnostics, pathological investigations, and preclinical drug screening. Three-dimensional bioprinting is advancing researchers towards more effective cancer treatments and improved patient outcomes.

## 2. Conventional Research Methods for Modeling Cancer Vasculogenesis

The development of new blood vessels within tumors or cancer vasculogenesis contributes to the growth, invasion, and metastasis of cancers. Conventionally, both in vitro and in vivo models—each with advantages and disadvantages—have been used to study cancer vasculogenesis. In vitro models typically use cultured epithelial and cancer cells to mimic the environment surrounding the tumor. These models enable high-throughput screening of possible anti-angiogenic drugs and controlled experiments [82,83,84,85]. However, they lack the intricacy of in vivo tumor microenvironments, including interactions with extracellular matrix elements and stromal cells. A well-known in vitro approach is the tube formation experiment, which involves growing endothelial cells on a layer of Matrigel, an extract of the basement membrane, to evaluate the potential of the cells to develop capillary-like structures. This evaluation does not effectively replicate the dynamic nature of tumor angiogenesis and needs to be simpler and more repeatable [72,86,87,88].

In contrast, in vitro and in vivo models involve the implantation of cancer cells into animal models, such as mice, to study tumor development and angiogenesis [89,90]. Although these models provide a more physiologically native tumor microenvironment, they are costly, time-consuming, and ethically challenging. Additionally, the interpretation of the data could be challenged by the host immune system’s potential to influence tumor development and angiogenesis [91,92]. The chick chorioallantoic membrane (CAM) is a widely used in vivo model in which tumors or factors produced by tumors are implanted into the highly vascularized CAMs of chick embryos. This model enables real-time imaging of angiogenesis and evaluation of tumor-induced neovascularization. However, it is not as complex as mammalian systems and can adequately mimic the biology of human tumors [88,93].

Despite these limitations, conventional methods have played a significant role in advancing our understanding of cancer vasculogenesis and in the development of anti-angiogenic therapies (Table 1). For example, studies using these models have led to the discovery of vascular endothelial growth factor (VEGF) as a critical regulator of tumor angiogenesis, which provides a method for developing VEGF-targeted therapies such as bevacizumab. In conclusion, although conventional methods for studying cancer vasculogenesis have produced important information on tumor angiogenesis, they have limited scalability, applicability, and complexity [83,94,95]. New technologies, such as 3D bioprinted organoid cultures and microfluidic devices, provide viable options for high-throughput, physiologically accurate cancer vasculogenesis modeling.

### 2.1. Study of Cancer Vasculogenesis through Patient-Derived Xenograft (PDX) Models in Conventional Research Methods

**Figure 2:**
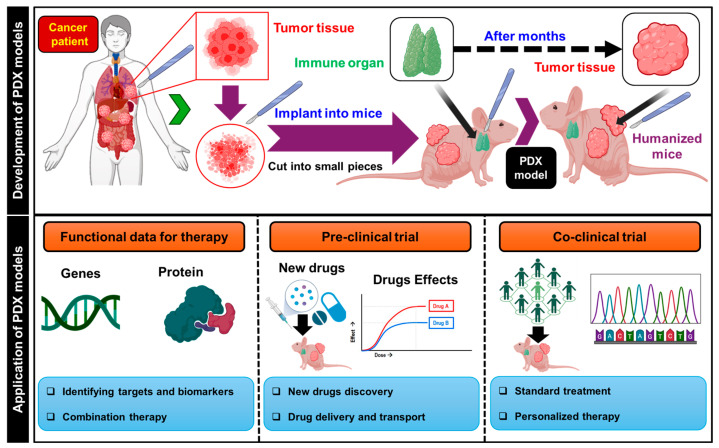
Schematic showing the development (**upper panel**) and application (**lower panel**) of patient-derived xenograft (PDX) models. These models were established by transplanting tumor tissue derived from patients into immunodeficient or humanized immune-deficient mice. Subsequently, they were utilized to explore the functionality of cancer vasculogenesis and to identify optimal therapeutic strategies.

To develop new targeted drugs, studying cancer vasculogenesis using patient-derived xenograft (PDX) models and conventional research methods is crucial. This approach allows for an in-depth examination of the intricate mechanisms associated with vascularization and cancer progression, providing vital insights for new treatment development. PDX models involve transplanting directly collected tumor tissues from patients into immunocompromised mice, enabling controlled laboratory development of the tumor microenvironment. Researchers can investigate cancer vasculogenesis mechanisms using conventional techniques such as immunohistochemistry, histological analyses, and genetic profiling. Figure 2 illustrates the diverse applications of PDX models in cancer vasculogenesis research.

Tumor vascularization facilitates cancer growth, proliferation, and metastasis by supplying essential nutrients. Disrupting tumor vasculature is crucial for inhibiting tumor progression. Deng et al. utilized cell-derived xenograft (PDX) models to enhance T cell infiltration and destroy tumor vasculature, aiming to increase CAR-T cell treatment efficacy. Combretastatin A-4 phosphate (CA4P) disrupts tumor vasculature by targeting its blood supply, leading to endothelial cell shrinkage and collapse. Deng et al. combined CA4P with HER2-CAR-T cell therapy, resulting in a significant reduction in tumor burden. The combination therapy effectively decreased tumor volume and prolonged survival, indicating a synergistic effect between CAR-T cells and CA4P treatment. Immunostaining targeting HER2 antigen revealed a reduction in HER2+ tumor cell count in the combined treatment group, suggesting its potential for enhancing immunotherapy efficacy and overcoming resistance mechanisms in cancer treatment [96].

**Figure 3:**
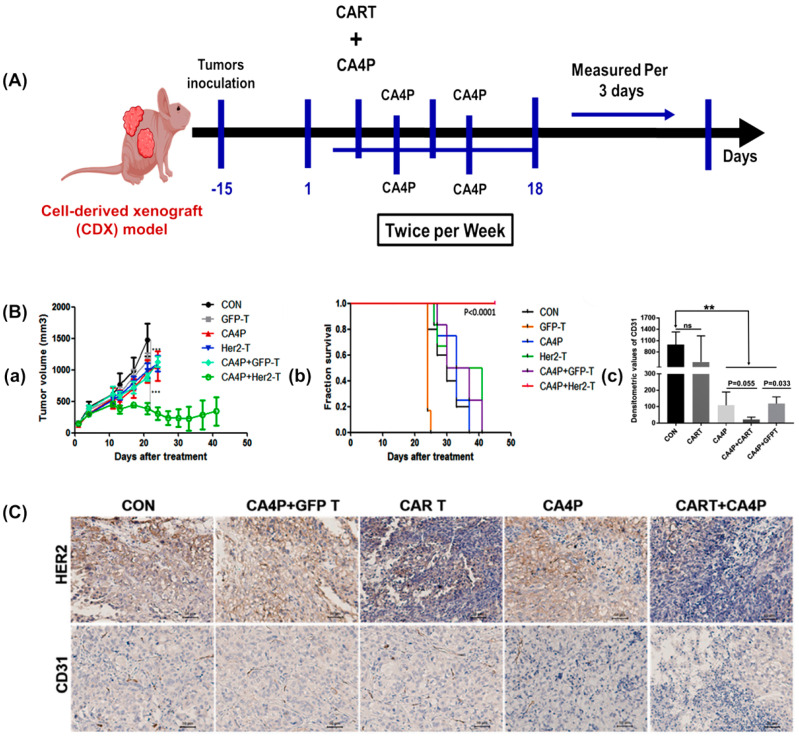
(**A**) The experimental setup involved establishing the SKOV3 cell-derived xenograft (CDX) model in nude mice. HER2-CAR-T cells were infused into the CDX model via the tail vein, alongside GFP-T cells. (**B**) (**a**) The results of the tumor growth curve in different experimental groups. (**b**) Kaplan–Meier survival analysis: the combination of HER-CAR-T cells with the CA4P group had a longer survival time compared to those in the other groups. ** *p* < 0.01, *** *p* < 0.001. ns indicates not significant. (**c**) The optical densitometric statistical analysis of the individual bands between the groups. (**C**) The effect of combination therapy on blood vessels and HER2 expression based on immunohistochemical analysis. Reproduced/adapted with permission [96].

This innovative strategy combines the vascular-disrupting drug CA4P with chimeric antigen receptor (CAR-T) cell therapy targeting human epidermal growth factor receptor 2 (HER2), offering a promising new direction in cancer treatment. Nude mice transplanted with SKOV3 cells were used to obtain tumors larger than 150 mm^3^. CA4P was administered via intraperitoneal injection prior to CAR-T or GFP-T cell infusion, with subsequent CA4P treatments administered twice weekly for a total of five sessions (Figure 3A). Effective vascular disruption was observed, leading to decreased tumor volume and increased survival time due to decreased expression of CD31 in the CA4P + CAR-T treatment group (Figure 3B). Immunohistochemical analysis confirmed the disruption of vascularization in this group (Figure 3C) [96].

Compared to no treatment, CA4P alone demonstrated some tumor growth inhibition, although this was not statistically significant. However, the combination therapy of HER2-CAR-T cells with repeated CA4P treatments notably decreased tumor volume and prolonged survival in experimental subjects. Immunostaining targeting the HER2 antigen revealed a significant reduction in HER2+ tumor cell count in the group receiving combined HER2-CAR-T cells and multiple CA4P injections compared to other treatment groups (Figure 3B,C) [96]. Therefore, CA4P and HER2-CAR-T cells hold potential for enhancing immunotherapy efficacy and eliminating resistance mechanisms in cancer treatment. 

This study investigates cancer-induced angiogenesis and vasculogenesis dynamics, endothelial cell proliferation, and molecular signaling pathways. It aims to improve cancer therapies, provide anti-angiogenic medications, and enhance our understanding of tumor biology. Significant advancements in oncology have been made in cancer vasculogenesis research, particularly with the application of PDX models and conventional methods. Researchers are developing novel techniques to ensure scientific integrity while addressing ethical concerns posed by animal models [97,98].

Patent-derived xenograft (PDX) and cell-derived xenograft (CDX) models have significantly advanced our understanding of cancer by replicating tumor heterogeneity and growth patterns. However, these models face challenges in studying cancer vasculogenesis as their vascular networks lack the complexity observed in human tumors. To overcome these limitations, researchers are utilizing 3D bioprinting methods to precisely design tumor microenvironments with physiologically realistic vascular networks. These 3D bioprinted models replicate complex interactions observed in human tumors by incorporating different cell types, extracellular matrix elements, and biochemical cues. Using 3D bioprinting to study cancer vasculogenesis can address the limitations of PDX and CDX models, facilitate more precise preclinical research, and accelerate the development of novel anti-angiogenic treatments [42,99].

### 2.2. Application of 3D Bioprinting in the Development of Cancer Vasculogenesis Models

Three-dimensional bioprinting has shown significant potential in medical oncology, particularly in developing cancer vasculogenesis models. These models aim to replicate the complex tumor microenvironment, including blood vessel development that is crucial for cancer growth and metastasis. By precisely controlling the vascular network architecture and content, researchers can explore various aspects of tumor angiogenesis and vasculogenesis using 3D bioprinting methods. Compared to conventional 2D cell culture, these models offer a more accurate representation of the in vivo tumor microenvironment by incorporating diverse cell types, extracellular matrix components, and biochemical signaling molecules [44,49,100,101].

Three-dimensional bioprinting methods provide researchers with a physiologically suitable platform to explore effective cancer treatments through the fabrication of complex models of cancer vasculature. The selection of bioinks, hydrogels containing growth factors, supporting cells, and extracellular matrix components, is crucial for success [102]. These bioinks are transformed into cancer models, allowing for the replication of the mechanical and biochemical cues that exist in vivo [103]. The precision of 3D bioprinting empowers researchers to replicate cancer vasculature within 3D bioprinted models by mirroring the dynamic nature of the cancer matrix through meticulous bioink composition customization (Table 2). 

**Figure 4:**
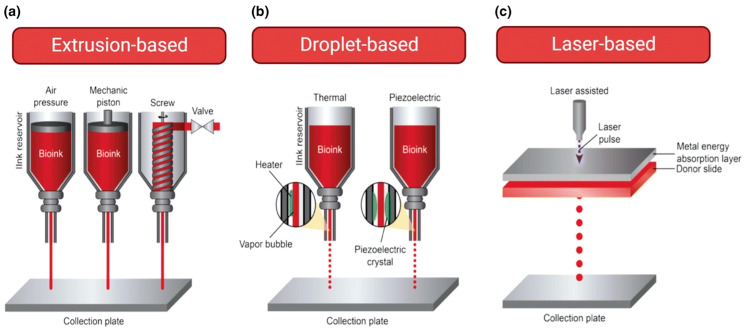
Different types of 3D bioprinter according to methods depositing bioinks: (**a**) extrusion-based bioprinter (EBB), (**b**) droplet-based, and (**c**) laser-based bioprinter. Reproduced/adapted with permission [109].

Vascular structure development in models is significantly possible by bioprinting technology, utilizing extrusion-based, droplet-based, and laser-based bioprinting techniques (Figure 4). Each bioprinting technique has advantages and disadvantages for fabricating vascular cancer models (Table 3). For instance, extrusion-based bioprinting (EBB) is an adaptable technology that allows for different viscosities of bioinks by extruding them via a nozzle utilizing mechanical or pneumatic actuators [105]. However, cell viability may be affected by increased extrusion pressure. While droplet-based bioprinting (DBB) provides precision by reproducing patterns with microscopic ink droplets, it has disadvantages associated with nozzle clogging and the selection of bioink viscosity. It is vital to preserve cell viability, especially in conditions with high shear stresses [106,107,108]. Laser-based bioprinting (LBB) presents a promising solution to the challenges of traditional bioprinting. Using laser-induced forward transfer or vat photo-polymerization, LBB offers high-resolution printing without the use of nozzles, thereby eliminating the issue of nozzle clogging. This technique also allows using bioinks with different viscosities and provides precision printing control. Despite its challenges, such as cost instruments, complex process parameters, and incompatibility with specific bioinks due to UV light toxicity and photoinitiator challenges, the personalized nature of LBB makes it a valuable tool in precision cancer vascular tissue development. 

As 3D bioprinting technology advances, particularly in bioink formulation and printing techniques, bioprinted tumor models become invaluable tools for understanding cancer biology and developing innovative treatments. Interdisciplinary collaboration and continuous innovation are crucial due to persistent challenges like scalability, precision, and validation [49]. The potential significance lies in using these models for drug testing and screening, offering a physiologically relevant environment to assess anti-angiogenic drugs and other treatments. By studying drug interactions with tumor vasculature within these models, researchers gain insights into treatment mechanisms and their effects on tumor development and metastasis. Moreover, personalized medicine methods targeting specific cancer patients become possible through the customization of these models with patient-derived cells. 

Vasculogenesis modeling using 3D bioprinting presents significant challenges and requires careful consideration of various factors. Replicating the complex structure and function of natural blood vessels within printed structures is particularly daunting due to their intricate network, varying diameters, branching patterns, and hierarchical organization. Precisely regulating bioink deposition, including vascular cells and supporting components, is crucial to address this challenge [13,125]. Introducing perfusable channels into the scaffold to facilitate nutrient and oxygen transportation is also essential for the survival and development of vascular tissues. Moreover, maintaining both high cell viability and mechanical stability of printed constructs poses additional challenges due to induced shear stress during the extrusion process and subsequent crosslinking techniques [126,127]. Despite these hurdles, the immense potential benefits of 3D bioprinting in promoting vascular cell proliferation, differentiation, and organization within printed structures are evident. By optimizing bioink formulations and printing conditions, these challenges can be overcome, paving the way for the development of vascularized tissue models. Once realized, these models will play invaluable roles in disease modeling, drug screening, and regenerative medicine, opening up new avenues for research and treatment. 

## 3. Advancements in 3D Bioprinting for Modeling Cancer Vasculogenesis

The combination of several different cell types, such as endothelial cells, pericytes, and cancer cells, in a spatially controlled manner is realized using 3D bioprinted cancer vasculogenesis models. This allows for rapid replication of the tumor microenvironment, improving drug responses and metastatic potential recommendations. These models can also be customized to replicate the specific characteristics of specific cancer subtypes, thereby allowing researchers to investigate the specific characteristics of different cancers and develop personalized treatment strategies. Therefore, it is very important to understand different approaches for developing vascular constructs. The most effective approach for creating vascular constructs is EBB, which utilizes further approaches in combination with direct and indirect extrusion methods. For mechanical integrity and cell viability, direct extrusion requires a precise deposition of bioinks containing hydrogels and cells, followed by solidification in a suitable environment. Cell survival is affected by challenges, including shear-induced pressures on cells during extrusion and its solidification methods. In this context, the work of Li et al. stands out for its novel approach to the development of vascularized liver tissue using dual nozzle extrusion bioprinting. This work underscores the importance of personalized hydrogel compositions and crosslinking techniques for achieving both short-term stability and long-term cell viability, marking a significant advancement in the field [128,129,130,131]. 

Dual-nozzle and coaxial extrusion methods, which provide enhanced control and precision, are the result of advancements in EBB technology. The concept of coaxial extrusion, where crosslinking occurs when the hydrogel and crosslinker solutions come into contact with one another, was pioneered by Ozbolat and colleagues. This method has revolutionized the field, as it now allows for the bioprinting of complex structures like perfusable conduits, showing remarkable structural integrity and cell survival. Moreover, the capabilities of EBB have been expanded by developments in microfluidic print heads, which enable the deposition of bioinks with different properties, such as gelMA and alginate, with different crosslinking kinetics, opening the possibility of developing more intricate and functional vascular structures [132,133]. 

The implications of these EBB advancements in tissue engineering and regenerative medicine are profound. EBB offers precision and versatility, capable of replicating complex vascular networks and developing personalized, self-organizing constructions. With further advancements in bioink formulations, crosslinking methods, and printing technologies, EBB has the potential to revolutionize the field. It could enable the development of vascularized tissues for various applications, including drug testing, disease modeling, and cell–cell interactions. This brings us closer to the ultimate goal of creating functional tissue constructs for medical applications [131,134,135].

**Figure 5:**
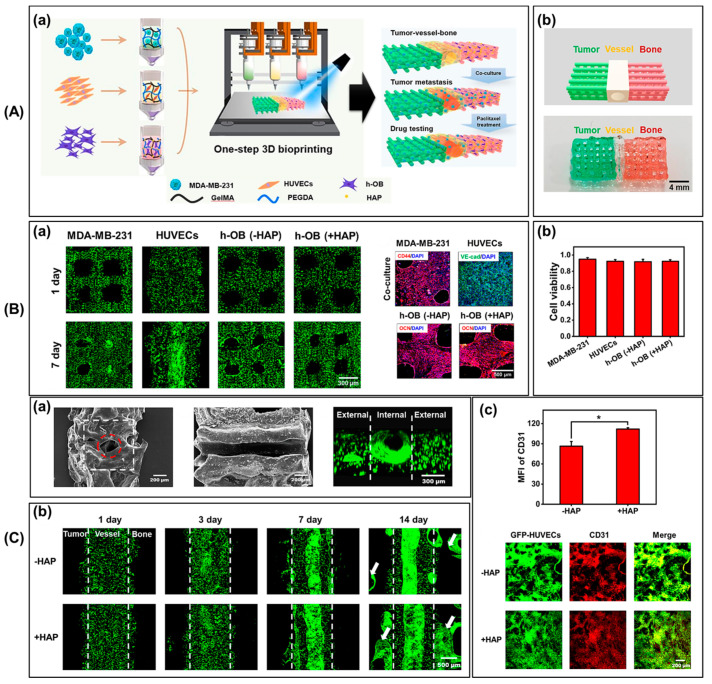
(**A**) Three-dimensional bioprinted breast cancer–vessel–bone model for drug testing. (**a**) Schematic of one-step 3D bioprinting for modeling the metastasis process and drug testing. (**b**) Computer-aided design and photograph of the co-culture model. (**B**) (**a**) Live/dead staining of cell-laden scaffolds cultured for 1 d and 7 d, along with immunostaining of relevant markers after 14 d of culture. (**b**) Cell viability within the co-culture model post-bioprinting. (**C**) Vascularization within the co-culture model. (**a**) Vascular channel morphology, distribution of green fluorescent protein (GFP), human umbilical vein endothelial cells (HUVECs), and immunofluorescence images [136]. The red circle indicates the internal cavity and the white dashed box outlines the entire vessel region. (**b**) The growth of GFP-HUVECs over 14 d. White dashed lines indicate the boundaries of the vessels, and white arrows point to vessels infiltrating the interior of the tumor and bone chambers. (**c**) Immunofluorescence images and mean fluorescence intensity (MFI) of CD31 after 14 d of co-culture. * *p* < 0.05. Reproduced/adapted with permission [136].

For example, Cheng et al. have pioneered the development of a groundbreaking 3D bioprinted scaffold capable of modeling breast cancer bone metastasis and facilitating drug testing. This innovative scaffold accurately mimics the behavior of native breast cancer cells, offering a biomimetic environment crucial for studying metastasis and testing drug efficacy. Shown in Figure 5(Aa), this novel structure faithfully replicates the behaviors of natural breast cancer cells in a biomimetic setting, which is essential for researching treatment responses and metastasis. Utilizing a one-step 3D bioprinting process, researchers developed a complex model comprising bone, vascular, and tumor tissues, as depicted in Figure 5(Ab). This efficient method allows for the simultaneous printing of multiple tissue types, facilitated by numerous printing channels and computer-aided design (CAD). Following a 7-day culture period, limited cell mortality was observed in the bioprinted co-culture scaffold, and all cell types exhibited significant growth from day 1, as indicated in Figure 5(Ba). Evaluation of cell viability, depicted in Figure 5(Bb), confirmed high survival rates post-bioprinting, demonstrating compatibility with cell viability [136]. Additionally, SEM analysis (Figure 5(Ca)) revealed the effective development of a vascular network resembling endothelial layers found in vivo. Subsequent confocal microscopy (Figure 5(Cb)) demonstrated the formation of endothelialized blood vessels within the scaffold, involving the precise alignment and integration of multiple cell types to mimic tissue angiogenesis and native vascular tissues. Evaluation of vascular network maturity via CD31 labeling showed a significant increase in CD31-positive cells, particularly in the co-culture group including hydroxyapatite (HAP), highlighting HAP’s role in vascularization (Figure 5(Cc)). 

By incorporating gelatin methacryloyl-based photocrosslinkable bioinks containing MDA-MB-231, HUVECs, and osteoblasts, researchers effectively reconstructed native metastatic niches and formed blood vessels and vascularized tissues within the scaffold. This model enhances our understanding of metastasis pathways by elucidating the dynamic invasive behavior of breast cancer cells and the interactions between cancer, vasculature, and bone cells. The conclusion of the bioprinted model of breast cancer–vessel–bone architecture represents a significant advancement in tissue engineering, with functional blood vessels expressing crucial markers like endothelial vascular CD31, cancer stem cell CD44, and osteogenic OCN. These markers are pivotal for the functionality of bioprinted vessels, ensuring vital characteristics such as barrier function and elasticity. Endothelial marker CD31 indicates an intact endothelium lining essential for barrier function, while CD44 indicates the model’s malignant potential, mimicking the complexities of the cancer microenvironment. The production of OCN highlights the model’s ability to replicate bone integration, which is crucial for studying cancer proliferation. This finding deepens our understanding of tumor–blood vessel interactions, presenting a promising platform for personalized medicine and drug testing that could revolutionize cancer therapeutic approaches [136].

Using 3D bioprinting technology, Lee et al. developed a microfluidic chip that replicates the complex tumor microenvironment (TME) of a self-assembled vascularized tumor. This innovative approach integrates self-organized TME arrays with a vascular endothelial barrier, overcoming challenges associated with traditional methods [137]. Vascular endothelial cells and breast cancer cells were induced into self-organization using a bioink composed of alginate and fibrin. The printing process involved swapping out the single printing nozzle for a 30-gauge single-barrel nozzle, along with the retention of the crosslinking aerosol tube to stabilize the printing of small amounts of bioink. Throughout the printing process, crosslinking aerosol was provided to enhance printability and reduce bioink evaporation. The printhead movement, guided by a G-Code for a 4 × 10 array, precisely transferred the bioink onto the microfluidic substrate. Crosslinking of the bioink occurred immediately following extrusion using a 10% CaCl_2_ aerosol. The comprehensive perspective of the complete process, from bioprinting to device fabrication, ensured replicated observations and experimental concentration distribution identical to the desired outcome. The fluid dynamics within the microfluidic substrate were characterized by observing the flow velocity inside the culture channel, which decreased rapidly as the liquid from the fluid distribution channels flowed into the larger culture channel. Evaluation of cell behavior based on the pillar gap revealed significant self-organization of BT474 cells and HUVECs within the TME construct. By the seventh day of culture, the TME construct exhibited complete self-organization, particularly in the group with the largest pillar gap of 150 μm. Confocal images at the liquid–hydrogel interface demonstrated the self-assembly of HUVECs to form a vascular barrier, while BT474 cells aggregated in the hydrogel area to form spheroids. The side view image on the YZ plane illustrated the attachment of HUVECs to the hydrogel wall and their coverage of the interface, while the Z-projection image depicted the arrangement of BT474 spheroids within the hydrogel construct, surrounded by an HUVEC barrier. This configuration was distinct from conventional systems where a TME construct with an HUVEC barrier surrounding BT474 spheroids was not observed. Through extrusion bioprinting on a microfluidic substrate, Lee et al. successfully developed a self-organized TME array-on-a-chip that enveloped breast cancer spheroids with a vascular endothelial barrier [137]. 

### 3.1. Pathological Study

The application of 3D bioprinting technology to cancer vasculogenesis models for pathological research provides an in-depth method for understanding the complexities of tumor pathology and development. The pathogenic characteristics of malignant vasculature may be studied under controlled and reproducible conditions because these models provide researchers with unique potential. A significant feature of this research is the characterization of the anatomical and functional properties of the vasculature found within the tumor microenvironment [138,139,140,141]. Using cutting-edge imaging techniques, including confocal and multiphoton microscopy, researchers can observe and study the complex networks of blood vessels, including their structures, densities, and permeabilities. The mechanisms associated with tumor angiogenesis and vascular remodeling, two essential steps involved in cancer development and propagation, are better understood through thorough pathological studies [140,141,142,143].

**Figure 6:**
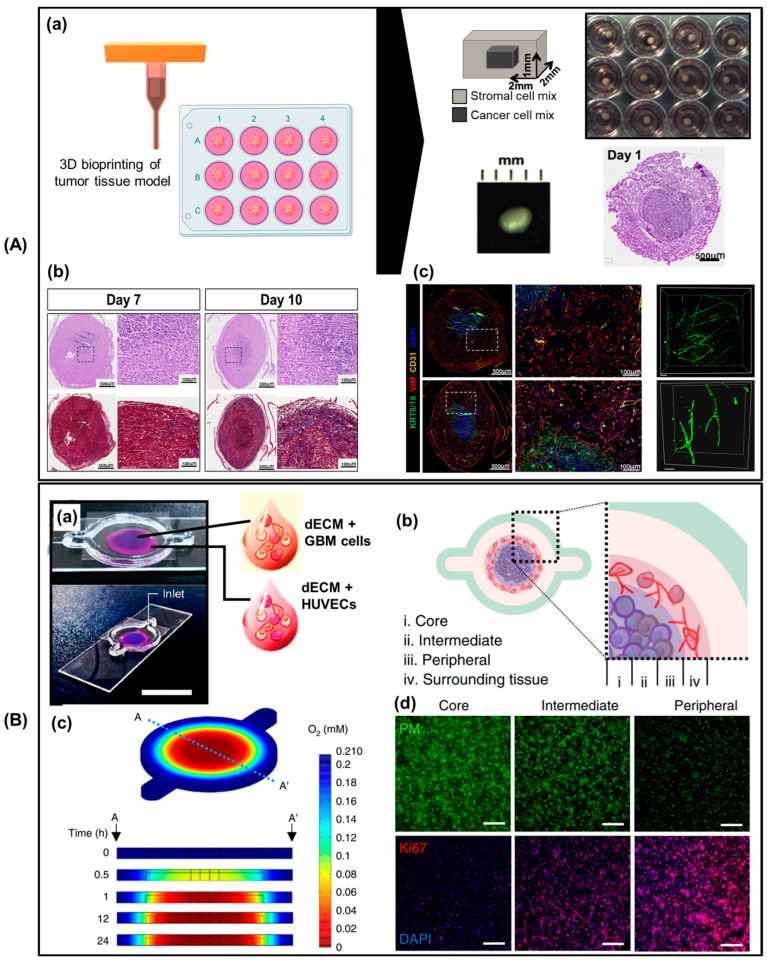
(**A**) Advances in 3D bioprinting for personalized pathological studies. (**a**) Schematic of a 3D bioprinted tissue cultured on transwells. (**b**) H&E and Masson’s trichrome images of bioprinted tumor tissues with MCF-7 and HUVECs in the cancer compartment and HMFs and HUVECs in the stromal compartment, cultured for 7 or 10 d. Each histological image on the right is a magnified view of the dashed box in the corresponding image. (**c**) Immunostaining results showing endothelial network in the engineered tissue. The white dashed boxes were magnified and shown on the right side of the respective results. KRT8/18: keratin 8/18, VIM: vimentin. Reproduced/adapted with permission [144]. (**B**) The development of glioblastoma (GBM)-on-a-chip through 3D bioprinting for personalized pathological studies. (**a**) Photographs showing a GBM-on-a-chip model with GBM cells (blue) or HUVECs (magenta) printed on gas-permeable silicone on a glass slide covered by a glass slip. (**b**) Compartmental subdivision into core, intermediate, and peripheral areas with GBM cells and an external layer with HUVECs. (**c**) Oxygen level simulation performed within the hydrogel. (**d**) Immunostaining images of the core, intermediate, and peripheral zones using pimonidazole for hypoxic cells, Ki67 for oxygenated proliferating cells, and DAPI for cell nuclei. Scale bar: 200 μm. PM: pimonidazole. Reproduced/adapted with permission [43].

For example, personalized patient therapy and diagnostics may be revolutionized by patient-specific ex vivo models of human malignancies that replicate the complex ecological and pathological characteristics associated with native tumors [144,145]. Gallego–Perez et al. examined the movement of patient-derived glioma, lung cancer, and colon cancer cells within tubule-like chip structures produced through laser-assisted prototyping [146]. The results showed that the tumor microenvironment affects tumor migration. Patient-derived pancreatic tumor cells, fibroblasts, and HUVECs were used by Langer et al. [147] to develop a scaffold-free model of tumor-stromal interactions utilizing a microextrusion bioprinter. This increased tissue maturation, organization, and matrix protein deposition (Figure 6A). The tumor measured 2 × 2 × 1 mm^3^ and was grown directly on a Transwell insert. Yi et al. [145] developed a segmented cancer–stroma concentric-ring construction using an extrusion bioprinter, glioblastoma tumor cells from patients, human umbilical vein epithelial cells, and acellular extracellular matrix components derived from brain tissue. They fabricated a model that could replicate native tumors with structural, biochemical, and biophysical characteristics, while maintaining a radial oxygen gradient. The ability of the 3D printing-based method to develop a glioblastoma-on-a-chip in an appropriate period (1–2 weeks) indicated by the results suggested that point-of-care testing in a clinical setting would be possible (Figure 6B).

These studies have highlighted the practical importance of in vitro tumor models, showing that it is feasible to develop personalized tumor models directly from patient cells. The difficulties with primary tumor cell culture, shortage of sample sources and preservation methods, viability of developing dynamic tumor models at different phases of the disease, and other challenges should not be neglected when considering the present challenges to the success of personalized model development. In addition, pathological analysis of 3D bioprinted models of cancer vasculogenesis facilitates the evaluation of cellular activities and interactions in the tumor microenvironment. Researchers may study the dynamic interactions between several components of tumor vasculature by combining different cell types, such as stromal, endothelial, and cancer cells. This includes studying how cancer cells migrate, proliferate, and invade the vascular network, as well as how supporting stromal cells migrate to the tumor site. To find effective methods for interrupting tumor vascularization to prevent tumor development, researchers can also evaluate how the tumor vasculature responds to medical therapies, such as immunotherapies or anti-angiogenic medications [43,140,148,149].

In addition, pathological examination of 3D bioprinted models of cancer vasculogenesis facilitates the study of disease-specific characteristics and biomarkers associated with different cancer types. Researchers can replicate the molecular and genetic factors associated with specific cancer subtypes using patient-derived tumor cells or modifications to genes in the model. This enables customized pathological examinations and drug evaluations. This method facilitates the identification of new biomarkers for early cancer diagnosis and prognosis and provides a platform for evaluating patient-specific targeted drugs. In conclusion, the pathological analysis of 3D bioprinted cancer vasculogenesis models holds great potential for improving the knowledge of tumor biology and creating more potent diagnostic and therapeutic approaches for cancer treatment [30,150,151].

### 3.2. Preclinical Drug Screening Platform for Personalized Medicine

**Figure 7:**
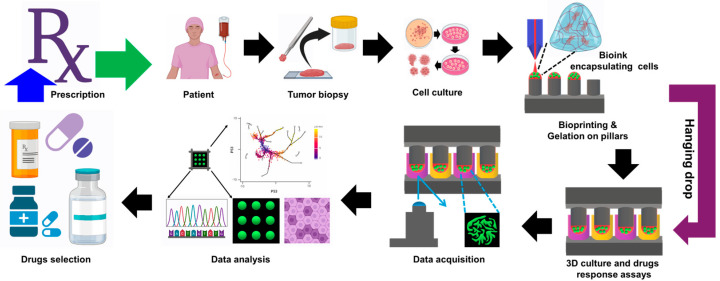
Schematic showing the design of a preclinical drug screening platform for personalized medicine by 3D bioprinting technology.

Three-dimensional bioprinting platforms have been utilized to screen drugs for pathological preclinical studies, which have been transformed into personalized medicine by creatively utilizing three-dimensional bioprinting technology. This cutting-edge technological method allows researchers to construct complex 3D models of human tissues that closely replicate the structures and functions of the natural organs in the body. More physiologically appropriate conditions for drug testing can be found in these bioengineered constructs than in conventional 2D cell cultures. Three-dimensional bioprinting allows the precise development of cancer-specific microenvironments that facilitate the assessment of medication toxicity and efficacy (Figure 7). This is achieved by precisely controlling the spatial arrangement of cells, extracellular matrix components, and biochemical signals [152,153].

The development of personalized therapies for specific patient requirements can be realized by incorporating 3D bioprinting into preclinical drug screening. Researchers can construct personalized disease models that closely mimic the pathological conditions observed in specific patients by utilizing the cells or tissues collected from patients. This approach allows the detection of patient-specific responses to different drugs and enhances the predictive value of preclinical research. These complex screening methods have significant potential for accelerating the research and development of safe and effective drugs, introducing them into the modern era of precision medicine [60,154,155].

**Figure 8:**
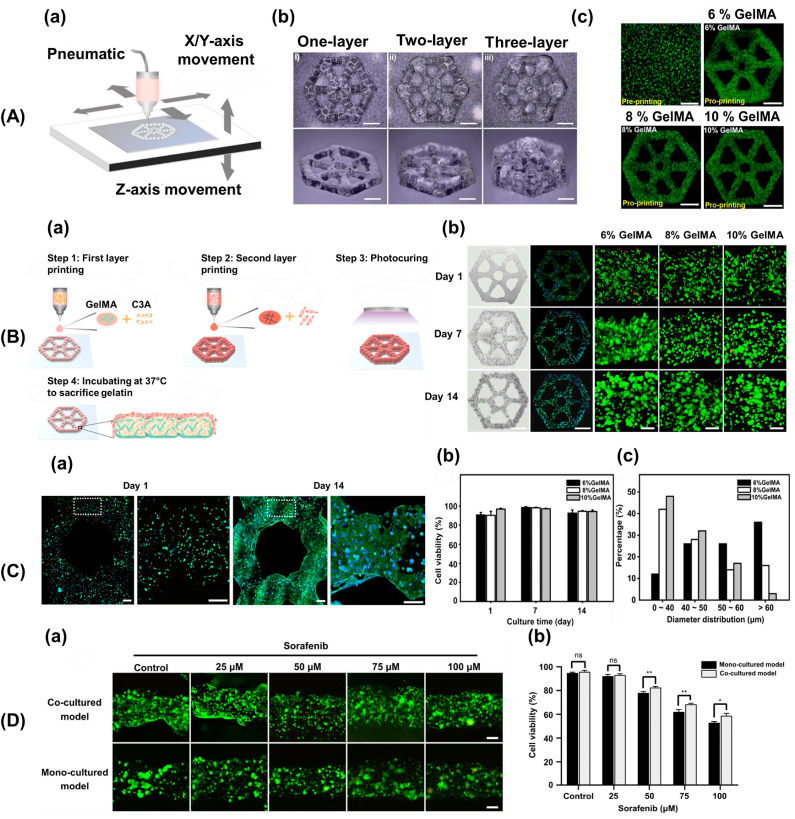
(**A**) Fabrication of liver lobule-like constructs. (**a**) Schematic depicting liver lobule-like structures produced via GelMA hydrogel beads by the dot extrusion printing system. (**b**) Images display layered lobule-like structures. Scale: 1 mm. (**c**) Live/dead analysis of C3A cells pre- and post-printing. Scale: 1 mm. (**B**) Endothelialized liver lobule-like constructs: (**a**) Schematic outlining four-step printing of endothelialized liver lobule-like constructs. (**b**) Bright-field and F-actin fluorescent images exhibit constructs on days 1, 7, and 14. Scale: 2 mm. Live/dead analysis of constructs with varied GelMA concentrations over 14 d. Scale: 200 µm. (**C**) In vitro evaluation: (**a**) F-actin staining reveals cell morphology at days 1 and 14. The white dashed boxes were magnified and shown on the right side of the respective results. Scale: 200 µm. (**b**) Cell viability at days 1, 7, and 14. (**c**) Cell diameter distributions after the 14-day cultivation with different GelMA concentrations. (**D**) Drug evaluation: (**a**) Live/dead images illustrate 3D liver cancer models post-sorafenib incubation. Scale: 200 µm. (**b**) Statistical analysis of cell viability in both constructs at varied drug concentrations. * *p* < 0.05, ** *p* < 0.01. ns indicates not significant. Reproduced/adapted with permission [156].

Fan et al. developed an innovative endothelialized liver lobule-like construct to investigate liver cancer, which is crucial for in vitro drug screening and disease mechanism analysis (Figure 8). Despite advancements, accurately mimicking native liver tumor structures in 3D models remains challenging. By leveraging dot extrusion printing technology, they produced precise hepatocyte-laden methacryloyl gelatin (GelMA) hydrogel microbeads and HUVEC-laden gelatin microbeads. This enabled the creation of liver lobule-like structures with adjustable scales [156]. A vascular network was established by sacrificing the gelatin microbeads, which facilitated HUVEC proliferation in the hepatocyte layer. Using these constructs, the authors conducted sorafenib drug screening, which revealed enhanced drug resistance compared to monocultured constructs or hepatocyte spheroids alone. The 3D liver cancer models accurately replicated liver lobule morphology, offering promise as a liver tumor-scale drug screening platform. This approach represents a significant advancement in the study of liver cancer mechanisms and screening for potential therapies [156].

Han et al. successfully biofabricated a vascular tumor microenvironment (TME) using 3D bioprinting methods. This was achieved by incorporating human vascular endothelial cells (HUVECs) and lung fibroblasts (LFs) into a gelatin/alginate/fibrinogen (GAF) hydrogel, which eventually formed blood vessels with lumens. The purpose of this was to create a platform for testing anti-cancer drugs such as temozolomide (TMZ) and sunitinib (SU) on MCTSs with and without the blood vessel layer. The potential of bioprinted hydrogel layers containing LFs and HUVECs to form blood vessels was evaluated by staining the nuclei with DAPI, the blood vessels with anti-CD31 antibody, and the actin with phalloidin [157].

The formation of microvessels is shown to be significant after the encapsulation of HUVEC/LF. The results demonstrated the formation of capillary networks by day four, showing the early development of endothelial junctions between cells in the capillary tube; however, the microvascular network needed to be completed in these images. By day seven, highly expressed CD31 and well-developed capillary networks were seen. The vessels on day seven are more prominent and thicker than those on day four, based on a quantitative examination of blood vessel area and thickness. Day seven of printing GAF hydrogel using HUVECs showed the development of microvessels, demonstrating the requirement of LFs for HUVECs to develop vascularized tissues. Vascularized tissue’s effects on MCTS growth, angiogenesis, and EMT. The MCTSs for the 3D development with vascularized tissues showed more prominent actin protrusions than those for the 3D construct without vascularized tissues, showing that the migratory activity of cancer cells was enhanced [35]. Neovascularized structures from the vascularized tissues penetrated into the MCTSs [157]. 

Vascularized multicellular tumor spheroids (MCTSs) were treated with the traditional anti-cancer medication temozolomide (TMZ), the blood vessel inhibitor sucralfate (SU), or a combination of the two to show the feasibility of employing vascularized tissues for drug testing. The MCTSs were treated with TMZ and SU either individually or in combination for three days starting on day zero. The diameters of the U87 MCTSs have been confirmed by Actin/DAPI staining. When comparing samples treated with SU or TMZ to those that were not, there was a significant reduction in the size of the MCTSs. Additionally, MCTSs treated with various medications showed significant variations in size. When TMZ and SU were combined, tumor size was further reduced than when either drug was used alone. These results are consistent with combination therapy results with TMZ and SU in mice implanted with U87 cells, suggesting a synergistic impact when both drugs are given simultaneously. The results in this microenvironmental context are consistent with those of in vivo studies. Moreover, the drug screening results could be deduced just from size because MCTSs of identical dimensions had originally been grown. Samples treated with isolinderalactone, an extract from the traditional Chinese herbal medicine Lindera aggregate, similarly reduced MCTS size. This shows that the bioprinted blood vascular layer is widely useful for testing different chemotherapy drugs and promotes interactions with several (about 4–5) MCTSs [157]. 

The heterogeneity observed in various types of cancer tissues with different vascular structures is a crucial component that affects the differences in drug sensitivity. Variations in treatment response are caused by the different mutations and phenotypic characteristics that cancer cells exhibit. In addition, the oxygenation levels, nutritional availability, and structure of stromal cells in the microenvironment surrounding cancer cells all affect the efficacy of the treatment. These complexities pose significant challenges in replicating cancer tissues, making the development of accurate and reliable 3D bioprinted models a pressing need in the field. The precision of bioprinted models in replicating the complex structure and function of natural tissues is a significant factor. The advancement of bioprinting technology has enabled the fabrication of more intricate vascular structures, yet effective biomimicry still presents challenges [158,159]. The accuracy with which the models replicate the physiological characteristics associated with specific cancer tissues can be influenced by changes in printing parameters, biomaterial selection, and cell sources. Therefore, enhancing drug screening outcomes and devising personalized medicine strategies hinge on refining bioprinting procedures to enhance the fidelity of vascularized cancer models [40,62]. 

In summary, the intricacy of cancer biology and the challenges in replicating it ex vivo are evident in the sensitivity of 3D bioprinted models with vascular structures to drug screens in various cancer tissues. Enhancing the accuracy and application of drug screening techniques necessitates tackling the inherent heterogeneity of cancer and enhancing the effectiveness of bioprinted models. By developing personalized experimental methods that incorporate these factors, researchers can effectively harness 3D bioprinting technology to expedite cancer research and drug development. 

### 3.3. Cancer Diagnosis

Bioengineered constructs provide effective platforms for drug screening, personalized treatment, and valuable knowledge regarding the mechanisms that control cancer growth. Three-dimensional bioprinting enables precise control of the spatial arrangement of cells and extracellular matrix components. This allows the recreation of complex tumor microenvironments, which advances the understanding of the interactions between tumors and hosts and supports the development of more efficient diagnostic and therapeutic methods.

**Figure 9:**
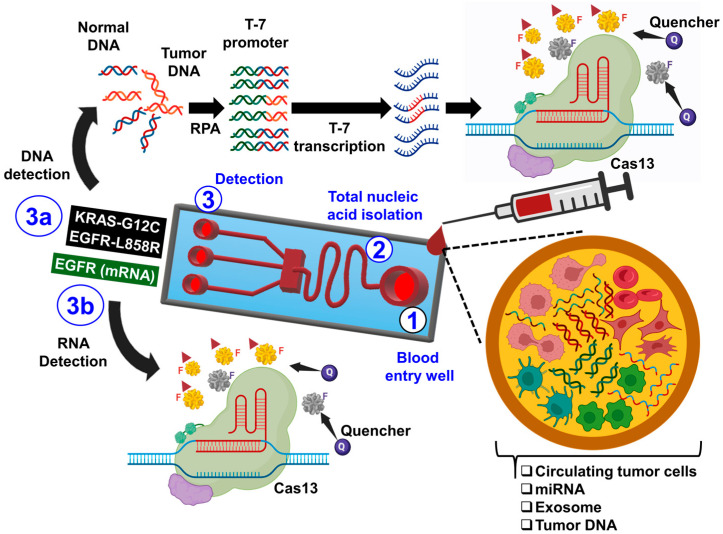
Schematic of a microfluidic biosensor utilizing Cas13 for cancer diagnosis. Blood samples from non-small-cell lung carcinoma (NSCLC) patients are directly inputted into the device. Nucleic acids are isolated and directed into wells containing reagents for detecting DNA mutations or quantifying overexpressed NSCLC-associated RNAs. Cas13 is activated by targeting RNAs, leading to the cleavage of target RNAs and fluorescent reporter RNAs. Tumor DNAs are amplified using recombinase polymerase amplification, followed by transcription with T7. Cas13 binds to mutation-containing transcripts, cleaving fluorescent reporter RNAs for signal detection.

Advancing affordable and rapidly clustered regularly interspaced short palindromic repeat (CRISPR)-based nucleic acid detection techniques exhibits promise for early cancer detection [160]. CRISPR systems are complex in prokaryotes and feature effectors primarily comprising Cas12 or Cas13 proteins guided by CRISPR RNA (crRNA) to target and cleave pathogenic nucleic acids [161,162]. Zhang et al. pioneered a specific, high-sensitivity, enzymatic reporter-unlocking method. This innovative approach combines recombinase polymerase amplification technology with the collateral single-stranded DNase activity of Cas13, enabling highly sensitive and specific detection of cancer-associated mutations in cell-free DNA, such as the EGFR-T790M and EGFR-L858R mutations observed in patients with non-small-cell lung carcinoma (NSCLC) [163]. Following liquid biopsy, the targeted mutations in cell-free DNA were identified using fluorescent and lateral flow-based readout methodologies, as shown in Figure 9.

**Figure 10:**
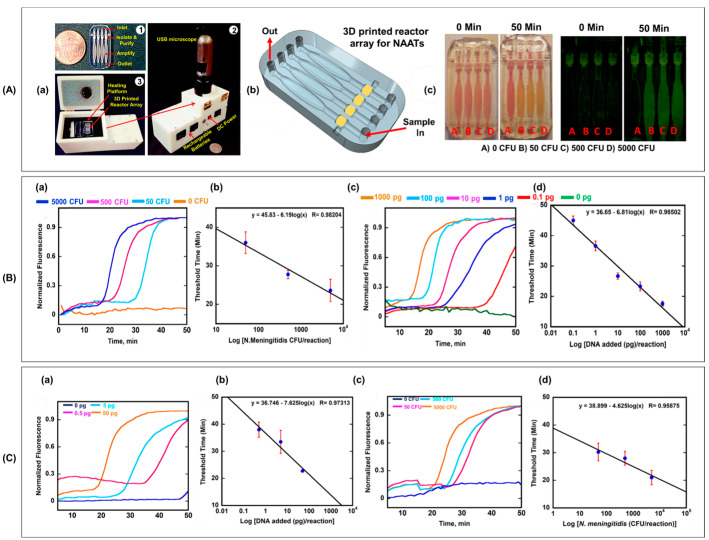
(**A**) (**a**) Portable heater module with a USB microscope and heat platform for a 3D-printed array facilitates loop-mediated isothermal amplification (LAMP). (**b**) Assembly schematic illustrating the multifunctional, leak-proof bonding of 3D printed reactor array for nucleic acid amplification tests (NAATs). (**c**) Representative photos showing a colorimetric LAMP assay detecting N. meningitides at different colony-forming units (CFU) per reaction. (**B**) (**a**) LAMP curves for P. falciparum exhibiting sensitivity from 0.1 to 1000 pg per reaction. (**b**) Calibration curve showing the relationship between log target concentration and amplification for P. falciparum. (**c**) LAMP curves for N. meningitidis ranging from 50 to 5000 CFU per reaction. (**d**) Calibration curve for N. meningitidis correlates log target concentration to amplification. WarmStart^®^ LAMP master mix was utilized. (**C**) (**a**,**b**) LAMP curves and calibration show quantitative detection of P. falciparum gDNA in plasma samples at spiked concentrations. (**c**,**d**) LAMP curves and calibration for quantitative detection of N. meningitidis bacteria in cerebrospinal fluid samples at varying concentrations. WarmStart^®^ LAMP master mix was employed. Reproduced/adapted with permission [164].

For microfluidic point-of-care diagnostics, Kadimisetty et al. demonstrated a recently introduced 3D-printed multifunctional microfluidic device that allowed them to extract, concentrate, and isothermally amplify nucleic acids in various body fluids, as shown in Figure 10. The microfluidic device was combined with a membrane to separate nucleic acids and then placed in a chamber that induced loop-mediated isothermal amplification [164]. Finally, the signal was detected colorimetrically using a smartphone and a USB fluorescence microscope. This indicates that the improvement of microfluidic diagnostic devices for low-cost, automated point-of-care applications through 3D printing has significant potential.

## 4. Limitation of Cancer Vasculogenesis Models

In cancer research, it is essential to understand the complex mechanisms involved in vasculogenesis, or the development of new blood vessels. However, current models of cancer vasculogenesis still have some distinct limitations, despite significant improvements [81,165]. The challenge of precisely replicating the tumor microenvironment is one of the significant difficulties. The dynamic interaction between tumor cells, endothelial cells, and the surrounding stroma in vivo is frequently missed by in vitro models, considering the advantage in that they provide controlled environments. As a result, the understanding of tumor angiogenesis and vasculogenesis that can be obtained from these models might need to be urgently revised, underscoring the importance of our work in this field.

One major limitation is that the heterogeneity in tumors cannot be replicated. Tumors are different because they contain different cell types with distinct genetic and phenotypic characteristics. Current models usually minimize this diversity, making implementing the findings in a clinical setting challenging [166,167,168]. This limitation underscores the urgency and importance of our work in addressing these issues. In addition, using mouse models explicitly presents several limitations, considering that they help understand tumor biology. The vascular structure of mice differs significantly from that of humans, which may impact the results and applicability of preclinical research [169]. Moreover, the temporal and spatial dynamics of tumor vasculogenesis are incredibly intricate, potentially surpassing the capabilities of the models in use today. Tumor blood vessel development is a highly regulated process affected by several components, such as growth factors, signaling pathways, and hypoxia. Simple models could be missing significant information in this process, which could result in inadequate or inappropriate conclusions. Moreover, the development of treatments that target certain stages of angiogenesis may be limited by the inability to replicate the temporal characteristics of tumor vasculogenesis [170,171,172].

## 5. Conclusions and Future Directions

Significant progress has been made in improving our understanding of tumor angiogenesis and microenvironment interactions, owing to the application of 3D bioprinting technology to cancer vasculogenesis models. Significant findings have provided information on the complex mechanisms underlying tumor vascularization, thus understanding tumor development, metastasis, and the response to treatment. This ability to precisely construct vascular networks in tumor models provides a more physiologically appropriate platform for research on tumor–host interactions, personalized medicine, and drug screening. Three-dimensional bioprinting has the potential to significantly contribute to research on vasculogenesis in cancer. Through precise replication of the complex structure and dynamics of the tumor vasculature, researchers may more effectively model the tumor microenvironment and explore the effects of various factors, including hypoxia, nutritional gradients, and immune cell infiltration. This enhanced understanding will ultimately improve patient outcomes by facilitating the development of customized anti-angiogenic drugs [44,139].

The application of 3D bioprinting technology in cancer research has propelled significant advancements in our comprehension of tumor angiogenesis with the interacted tumor microenvironment. This advancement has revealed complex mechanisms regulating tumor vascularization, such as the role of VEGF and angiopoietin in promoting angiogenesis and the influence of pericytes and endothelial cells in stabilizing blood vessels. These insights provide a deeper understanding of tumor initiation, metastasis, and therapeutic response. Researchers can now investigate tumor–host dynamics, personalized medicine, and drug screening from a more physiologically appropriate platform because of the efficient development of vascular networks within tumor models. The potential of 3D bioprinting for investigating cancer vasculogenesis is truly remarkable. Researchers can better model the tumor microenvironment to investigate the effects of different factors like hypoxia, nutritional gradients, and immune cell infiltration by precisely modeling the complex architecture and dynamics of the tumor vasculature. This will ultimately revolutionize the development of personalized anti-angiogenic therapies [40,173,174]. 

However, despite these promising developments, there are still significant challenges with the 3D bioprinting techniques used today for modeling the vasculogenesis of cancer. These challenges, including vascular perfusion, the emulation of various cancer vasculatures, and the spatially regulated incorporation of numerous cell types, are not to be underestimated. Furthermore, cost-effectiveness, repeatability, and scalability are essential components in ensuring the effective application of these models. Therefore, to enhance the practical significance of 3D bioprinting cancer vasculature models, future studies must urgently focus on overcoming these challenges and enhancing the technique. More advancements in printing processes, bioink formulations, and vascularization procedures will be essential for developing more functional and biomimetic tumor models. In addition, using modern imaging modalities like microfluidics and multiphoton microscopy can allow for real-time monitoring of the development of tumors and the efficacy of treatments in these models. The challenges we face are significant, but with determination and innovation, we can overcome them and revolutionize cancer research [16,57].

Therefore, 3D bioprinting is revolutionizing the discipline of cancer research by providing cutting-edge tools for investigating the complex mechanisms associated with tumor vasculogenesis. This is not a journey we can embark on alone. Interdisciplinary association with continuous innovations is essential if personalized medicine and cancer medicines are to achieve their maximum potential. It can accelerate the development of personalized treatment options by identifying novel perspectives into cancer biology and overcoming current limits by continuously improving technology. Significant advancements in the battle against cancer will be made possible by combining various areas of knowledge and a solid commitment to innovation in this collaborative effort. Together, we can make a difference in the fight against cancer. In summary, 3D bioprinting has significant potential for transforming the field of cancer research by offering advanced platforms for investigating tumor vasculogenesis. To overcome existing limitations and fully utilize this technology to advance cancer therapies and personalized medicine, multidisciplinary collaboration and continuous innovation are essential.

## Figures and Tables

**Table 1:** Conventional methods for studying cancer vasculogenesis.

Method	Advantages	Limitations
In vitro tube formation assay	- Simple and reproducible. - Allows for high-throughput screening of potential anti-angiogenic agents.	- Lack of physiological relevance, as it does not fully mimic the tumor microenvironment. - Unable to replicate the dynamic nature of tumor angiogenesis.
In vivo chick CAM assay	- Provides a physiologically relevant environment for studying tumor angiogenesis. - Allows for real-time visualization of angiogenesis. - Assessment of tumor-induced neovascularization.	- Time-consuming and expensive. - Ethically challenging, as it involves the use of animal models. - The immune system of the host animal may influence tumor progression and angiogenesis, complicating data interpretation.
Xenograft mouse models	- Recapitulates human tumor biology more closely. - Allows for assessment of tumor growth and angiogenesis in vivo. - Enables evaluation of the efficacy of anti-angiogenic therapies.	- Time-consuming and expensive. - Ethically challenging, as it involves the use of animal models. - The immune system of the host animal may influence tumor progression and angiogenesis, complicating data interpretation.
Patient-derived xenograft models	- Enables evaluation of tumor response to therapy in a preclinical setting. - Allows for personalized medicine approaches by testing patient-specific treatments.	- Time-consuming and expensive. - Ethically challenging, as it involves the use of animal models. - May not fully recapitulate tumor microenvironment due to the absence of the human immune system. - Variability in engraftment rates and tumor growth kinetics.

**Table 2:** Polymers commonly used in the printing of vascular structures construction [104].

Biomaterial	Advantage	Disadvantage	Application
Gelatin	Excellent biocompatibility, good cell adhesion, physical crosslinking properties	Low shape fidelity, especially unstable at temperatures suitable for cell growth, and low mechanical strength	Modification such as methacryloyl anhydride, or cross-linking, enhances its mechanical strength and printing resolution
PU	Excellent histocompatibility, super mechanical strength	Cells cannot be encapsulated directly	3D printing vascular networks, bioartificial liver manufacturing
PLGA	Poor biocompatibility, middle mechanical properties	Cells cannot be encapsulated directly	3D printing vascular networks, bioartificial liver manufacturing
Alginate	Shear thinning properties, very short time polymerizable, porous properties	Poor biocompatibility, low cell adhesion properties	Often mixed with gelatin, hyaluronic acid, etc. for printing; as a sacrificial material for vascular stents
Fibrinogen	Excellent biocompatibility, good cell adhesion	Low mechanical strength, fast degradation rate	Commonly used for thrombin cross-linking, blending or double cross-linking with gelatin, sodium alginate, etc.
Hyaluronic Acid	High water absorption, excellent biocompatibility, low molecular weight has the ability to promote cell proliferation	Low mechanical strength and poor formability	Modification such as methacryloyl anhydride, or compounded with other materials
dECM	Promotes cell adhesion, proliferation and functionalization, especially has a certain antithrombotic effect	Low mechanical strength, slow gelation, complicated preparation process	Often used with fast cross-linking materials such as sodium alginate
Pluronic^®^ F127	High resolution printing, special temperature sensitive properties	Low mechanical strength, fast degradation rate	As a sacrificial material for vascular stents

**Table 3:** A comparative analysis of the advantages and disadvantages of extrusion-based, droplet-based, and laser-based bioprinting technologies.

Bioprinting Techniques	Advantages	Disadvantages	Outcomes	References
1. Extrusion-Based	- Ability to print a wide range of biomaterials. - High cell viability. - Cost-effective. - Ability to print large-scale tissues. - Precise control over material deposition. - Ability to create scaffolds with channels for nutrient and oxygen diffusion to enhanced angiogenesis.	- Limited resolution. - Difficulty in printing complex structures. - Prone to clogging. - Mechanical stress on cells. - Limited control over microenvironment. - Limited resolution may hinder precise control over vessel networks.	- Creation of complex vascular networks with varying vessel diameters and branching patterns. - Establishment of perfusable blood vessels within tumor constructs. - Investigation of cancer metastasis mechanisms through the study of vascularized tumor models. - Evaluation of anti-angiogenic therapies and their effects on tumor angiogenesis. - Assessment of therapeutic efficacy of chemotherapy drugs in vascularized tumor microenvironments.	[105,110,111,112,113,114,115,116]
2. Droplet-Based	- High resolution - Ability to print multiple materials simultaneously. - Minimal material wastage. - Capability to create complex structures. - High resolution allows for precise patterning of vascular structures, promoting angiogenesis.	- Limited range of printable materials. - Shear stress during droplet formation. - Difficulty in maintaining cell viability. - Limited scalability. - Issues with droplet coalescence and satellite droplets. - Shear stress during droplet formation may affect cell viability.	- Formation of functional blood vessels within tumor tissue. - Enhanced tumor growth and invasion due to the presence of functional blood vessels. - Accurate recapitulation of tumor microenvironment, aiding in drug screening and personalized medicine. - Improved understanding of tumor angiogenesis processes.	[106,117,118,119,120]
3. Laser-Based (LBB)	- High resolution. - High printing speed. - Precise control over spatial positioning. - Minimal mechanical stress on cells. - Ability to create intricate microstructures. - Precise control over spatial positioning enables the creation of intricate vascular networks.	- Limited range of printable materials. - Expensive equipment and maintenance. - Potential phototoxicity. - Limited scalability. - Complexity in optimizing printing parameters. - Limited range of printable materials may affect the mimicry of natural tissue environments.	- Improved understanding of tumor angiogenesis processes. - Formation of intricate, high-fidelity vascular networks. - Simulation of tumor growth and invasion dynamics in a controlled in vitro environment. - Investigation of tumor-stromal interactions and their role in tumor progression and metastasis. - Assessment of therapeutic responses in the presence of functional blood vessels, mimicking in vivo tumor microenvironments. - Development of personalized cancer treatment strategies by studying patient-specific tumor responses in vascularized tumor models.	[112,121,122,123,124]

## Data Availability

Data sharing is not applicable.
